# Case of Diseased Knee-Joint, with Amputation of the Thigh

**Published:** 1829-04

**Authors:** Samuel Williams Jewel

**Affiliations:** Assistant Surgeon to H. M. Ship Warspite, Plymouth.


### DISEASED KNEE-JOINT.

*Case of Diseased Knee-Joint, with Amputation of the Thigh.* By Mr. Samuel Williams Jewel, Assistant Surgeon to H. M. Ship Warspite, Plymouth.

Thomas Soloman, ætatis fourteen, received a severe blow in the right knee, which caused immediate and extensive inflammation in the joint. A practitioner was sent for, who employed the usual means adopted in similar cases, such as the application of leeches, poultices, purgatives, and ultimately blisters, without so much reduction of the swelling as had been anticipated. The parents of the boy, finding that the swelling did not subside, consulted a quack of notorious celebrity for the cure of diseased joints, who, after using a variety of remedies for a period of five months, recommended the application of a poultice composed in part of quick lime, that, as he stated, a discharge might be produced, which would tend much to the recovery of the limb.

Having at this time been requested to see the patient, I found the knee enormously enlarged, measuring in circumference seventeen inches, and the tumor occupying nearly two-thirds of the thigh. Finding the tumor to have assumed a highly malignant character, with great constitutional derangement, I advised amputation of the limb without loss of time, which was consented to two days afterwards. The operation was performed as follows:

Having requested an assistant to compress the artery at the groin with his thumb, I commenced the operation in the usual way, by making a circular incision through the integuments, above five inches below Poupart's ligament, dissecting them from the superficial layer of muscles, and everting them about three inches; a second incision was made, commencing at the base of the integuments, dividing the first layer of muscles, and afterwards the deeper seated, carefully denuding the bone, which was sawed through. The femoral, ischiatic, and four small perforating arteries were secured, and the edges of the wound brought together. The integument and a large cushion of muscle formed a good covering for the bone.

The healing of the stump was rapid, and the boy was able to sit up between the second and third week. Five of the ligatures came away on the twelfth, and the other, from the femoral artery, on the fifteenth day.

The removed limb was found to weigh thirty-five pounds, notwithstanding a quantity of serum had escaped. Upon opening the joint, about an ounce and a half of synovial fluid escaped; and, upon close examination, I discovered a large excrescence of fungoid bone, arising by a small pedicle from the inner and upper surface of the inner condyle, and a smaller one on the outer. The crucial ligaments and semilunar cartilages were displaced from their original situations, and the reflected synovial membrane was much thickened and considerably inflamed. The tibia had also participated in the disease, from the numerous spiculæ of bone projecting into the cavity of the joint. The periosteum was found detached half way up the shaft of the bone; and the bone itself, when sawed through longitudinally, was found to be much more cancellated than natural.

*February 24th,* 1829.

